# DO MINDFULNESS ACTIVITIES IMPROVE HANDGRIP STRENGTH AMONG OLDER ADULTS: A PROPENSITY SCORE MATCHING APPROACH

**DOI:** 10.1093/geroni/igae098.3252

**Published:** 2024-12-31

**Authors:** Muhammad Thalil, Waad Ali, Boadi Agyekum, Shobhit Srivastava

**Affiliations:** The Pennsylvania State University, University Park, Pennsylvania, United States; Sultan Qaboos University, Muscat, Masqat, Oman; University of Ghana, Acra, Greater Accra, Ghana; International Institute for Population Sciences, Mumbai, Maharashtra, India

## Abstract

The integration of mindfulness practices into the daily routine of the elderly population has been proven to enhance their overall well-being and vitality. There is limited evidence regarding the association between mindfulness activity and physical strength among community-dwelling older adults. Since hand grip strength is an important measure for evaluating the health and well-being of older adults, we explored the association between yoga/mindfulness practices and handgrip strength among older Indians using nationally representative data (N=27,707; mean age: 68.6 [SD:7.3]). Handgrip strength was assessed using a handheld Smedley Hand Dynamometer and mindfulness activities were self-reported. Key findings showed that for the matched sample (ATT), handgrip strength increased by 0.55 points for those who were involved in mindfulness activities compared to older adults who were not involved in mindfulness activities. The results of the Average Treatment Effect on the Untreated (ATU) showed that among individuals who were not involved in mindfulness activities, if they were involved in mindfulness activities, their handgrip strength was likely to increase by 1.16 points. The findings of the current study highlight the importance of integrating mindfulness techniques, including yoga, into public health programs aimed at ageing populations. By tailoring personalized programs that improve both mental and physical health, older adults can maintain an active lifestyle, thereby reducing healthcare costs and enhancing societal well-being.

